# The economic burden of cervical cancer from diagnosis to one year after final discharge in Henan Province, China: A retrospective case series study

**DOI:** 10.1371/journal.pone.0232129

**Published:** 2020-05-07

**Authors:** Qianhui Wu, Manman Jia, Hongmin Chen, Shaokai Zhang, Yang Liu, Kiesha Prem, Mengcen Qian, Hongjie Yu

**Affiliations:** 1 School of Public Health, Fudan University, Shanghai, China; 2 Henan Cancer Hospital, Affiliated Cancer Hospital of Zhengzhou University, Zhengzhou, China; 3 Department of Infectious Disease Epidemiology, London School of Hygiene and Tropical Medicine, London, England, United Kingdom; 4 Key Laboratory Health Technology Assessment (Fudan University), Ministry of Health, Shanghai, China; 5 Key Laboratory of Public Health Safety (Fudan University), Ministry of Education, Shanghai, China; Chinese Academy of Medical Sciences and Peking Union Medical College, CHINA

## Abstract

**Background:**

In China, the disease burden of cervical cancer remains substantial. Human papillomavirus (HPV) vaccines are expensive and not yet centrally funded. To inform immunization policy, understanding the economic burden of the disease is necessary. This study adopted a societal perspective and investigated costs and quality of life changes associated with cervical cancer from diagnosis to one year after final discharge in Henan province, China.

**Methods:**

Inpatient records of cervical cancer patients admitted to the largest cancer hospital in Henan province between Jan. 2017 and Dec. 2018 were extracted. A telephone interview with four modules was conducted in Jun.-Jul. 2019 with a 40% random draw of patients to obtain direct non-medical costs and indirect costs associated with inpatients, costs associated with outpatient visits, and changes in quality of life status using the EQ-5D-5L instrument. Direct medical expenditures were converted to opportunity costs of care using cost-to-charge ratios obtained from hospital financial reports. For each clinical stage (IA-IV), total costs per case from diagnosis to one year after final discharge were extrapolated based on inpatient records, responses to the telephone interview, and recommendation on outpatient follow-ups by Chinese cervical cancer treatment guidelines. Loss in quality-adjusted life years was obtained using the ‘under the curve’ method and regression predictions.

**Results:**

A total of 3,506 inpatient records from 1,323 patients were obtained. Among 541 randomly selected patients, 309 completed at least one module of the telephone interview. The average total costs per case associated with cervical cancer from diagnosis to one year after final discharge ranged from $8,066-$22,888 (in 2018 US Dollar) and the quality-adjusted life years loss varied from 0.05–0.26 for IA-IV patients.

**Conclusions:**

The economic burden associated with cervical cancer is substantial in Henan province. Our study provided important baseline information for cost-effectiveness analysis of HPV immunization program in China.

## 1. Introduction

The disease burden of cervical cancer has been substantial globally, especially for less developed regions. In 2018, there were approximately 570,000 new cases and 311,000 cervical cancer related deaths, more than 84% of which were from low- and middle-income countries (LMIC) [1]. In China, cervical cancer is the second most common female malignancy and a leading cause of cancer-related death among women. It is estimated that in 2015 cervical cancer caused 30,500 deaths and recorded 98,900 new cases [2]. From 2000 to 2014, crude incidence rates of cervical cancer increased by 10% and 12.5% annually in urban and rural China, respectively [3].

Persistent human papillomavirus (HPV) infection is a necessary cause of cervical cancer [4]. If left undetected or untreated for years or decades, cervical intraepithelial neoplasia may progress to cervical cancer [5]. Fortunately, prophylactic vaccines that can effectively prevent HPV infections have been licensed in many countries throughout the world, including China. To eliminate cervical cancer globally, the World Health Organization recommends HPV vaccination for all adolescent girls aged between 9–14 years as the primary target population and, if proved to be cost-effective, older females or males as the secondary target populations [6].

However, HPV vaccines are not currently centrally funded in China–individuals have to pay the full amount of the shots out-of-pocket. The total costs of three doses of HPV vaccines are $140-$444 (depending on the choice of vaccine and including the newly approved domestic bivalent HPV vaccine), corresponding to around 3.3% to 10.4% of the annual disposable income per capita in China as of 2018, which is very expensive for most to afford. To support the decision of introducing HPV vaccines into China’s National Immunization Programme, evidence based on cost-effectiveness analyses is necessary. Estimates of economic burden of cervical cancer in China provide important baseline data for such analyses.

Following up cervical cancer patients prospectively over the entire treatment process since diagnosis is an ideal study design. However, it is hard to implement due to the long treatment duration of cancers. Previous studies that examined economic costs associated with cervical cancer in China mainly adopted a hospital-based design and focused solely on total medical expenditures of inpatient stays [7–9]. Shi et al. and Liu et al. are two notable exceptions–they examined the direct costs of cervical cancer screening, diagnosis, and treatment using a micro-costing approach based on clinical pathways [10, 11]. However, the treatment process of cervical cancer can be complicated and include multiple inpatient admissions. Income losses and transportation costs associated with the disease also aggravate household-level financial burden. Moreover, although researchers have explored the health related quality of life (HRQoL) status of cervical cancer patients [12–14], the loss in quality-adjusted life years (QALY) associated with the disease in China is less well understood.

In this study, we adopted a societal perspective, extracted inpatient records, and conducted a retrospective telephone survey among cervical cancer patients who attended the largest cancer hospital in Henan province to estimate the economic burden of cervical cancer. We considered both direct and indirect costs from diagnosis to one year after final discharge. We also assessed the quality of life impact of cervical cancer using QALY loss.

## 2. Material and methods

### 2.1. Study design and patient enrollment

Henan province is located in central China, with a per-capita GDP level ranking the 18^th^ out of all 31 provinces in 2017. During 2011–2015, the central region of China accounted for 31% of total hospitalization expenditures for tumor treatment across the country, and specialized cancer hospitals amounted to 50% of the total [15]. The study hospital, Henan Cancer Hospital, is located in the capital city of Henan province. It is the largest hospital specializing in tumor treatment in the city and attracts a large number of patients with cervical diseases from surrounding areas.

**Fig 1:**
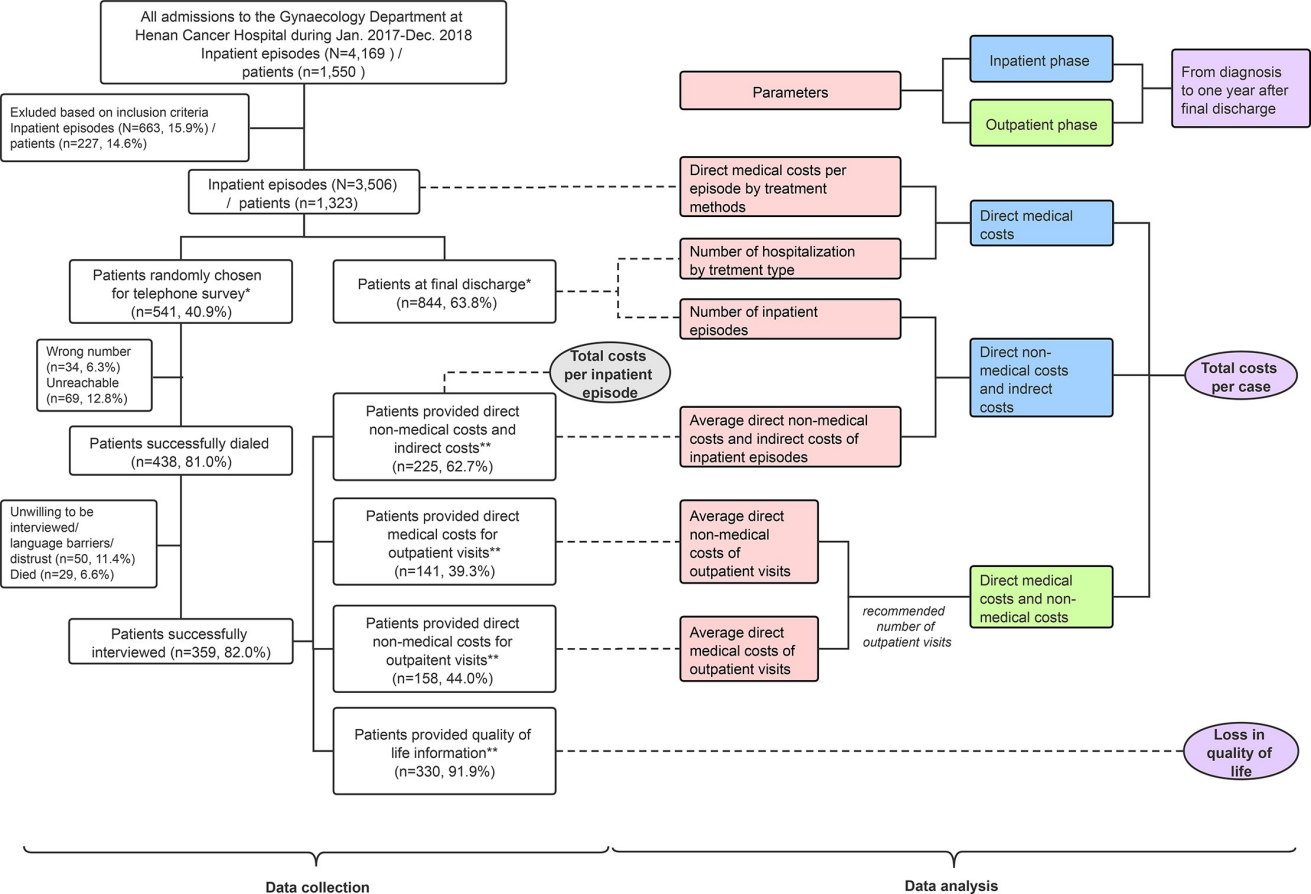
Conceptual diagram of data collection and analysis processes. After inclusion criteria were applied, patients experienced final discharges were identified (n = 844) and then a 40% random sample of all eligible patients was drawn. The two samples that labelled with * contained overlapping patients. During the telephone survey, samples were generated based on complete responses to each module. The four samples that labelled with ** also contained overlapping patients.

Patients who were admitted to the Gynecology Department at Henan Cancer Hospital between January 1, 2017 and December 31, 2018 were considered the potential participants of this study. Fig 1 provides an overview of the data collection and analytical processes. The left half of Fig 1 presents the patient enrollment procedure. Records of inpatient episodes from hospital information system for patients who met with the following inclusion criteria were extracted: (1) aged 18 years or above, (2) had been histologically confirmed of cervical cancer (ICD-10-C53), (3) had identified a definite clinical stage (IA-IV), (4) had received at least one type of treatment (i.e. surgery, radiotherapy, chemotherapy, or any two or three combination of the above) during hospitalization, (5) had no other cancers or precancerous conditions, and (6) were not patients with cancer relapse. These inpatient records contained clinical characteristics and medical expenditures (also referred to hospital charges, the total amount paid by the patients and the insurer) as well as demographic information of the patients.

Then, patients who completed inpatient treatment and were recommended for regular outpatient follow-ups were identified based on discharge summaries. Information on the entire treatment process of the patients was obtained based on treatment history on their last observed inpatient records.

Finally, a 40% random sample without replacement of all patients with inpatient records were invited to a telephone survey, where information on socioeconomic characteristics (education level and monthly family income) were collected. Patients were asked to recall costs associated with cervical cancer from diagnosis to the interview day and changes in quality of life states.

### 2.2. Telephone survey

The telephone survey was conducted between June 3, 2019 and July 25, 2019 by LinkDoc, a company specializes in patient follow-ups in China. Staffs from LinkDoc were trained and supervised by the research investigators. A computer-assisted telephone interviewing system was used to record answers directly into a computer. A patient was classified as unreachable if we failed to get in touch with her after four attempts made on different days. After verbal consents from the participants, demographic and clinical information was verified using their most recent inpatient records.

There were four modules in the survey. Accordingly, four samples were generated based on complete responses to each module. First, the EQ-5D-5L instrument was used to assess the health states of the patients. EQ-5D is a standardized five-dimensional preference-based health-related quality of life questionnaire designed by the EuroQol Group, which consists of a descriptive system with five dimensions and a visual analogue scale (VAS). The instrument has been validated in China and among cervical cancer patients in Asian populations [16, 17]. Patients were asked to report their self-rated level of health on the interview day and the day that they felt the worst since diagnosis to the interview day.

Second, non-medical costs associated with inpatient episodes were collected. Average direct non-medical costs (i.e. costs of transportation, accommodation, additional nutrients and nursing incurred to patients and their caregivers) and indirect costs (income losses incurred to the patients and caregivers) associated with each inpatient episode were obtained. For respondents who could not provide a detailed value for income loss, changes in employment status and loss of work days associated with inpatient episodes were asked and used as proxies.

Third, average direct medical expenditures (including both out-of-pocket and reimbursable portions) associated with each outpatient visit were obtained. Fourth, average direct non-medical costs associated with each outpatient visit were acquired. Average indirect costs associated with outpatient visits were not collected because most participants thought the amount was trivial. Indirect costs due to productivity loss and premature death were not considered.

### 2.3. Data analysis

The right half of Fig 1 summarizes the data analytical process. In China, prices of drug and healthcare services at public hospitals are determined by the government. Accordingly, medical expenditures may well represent the financial burden that incur to households and health insurance payers, but are not necessarily matched with costs incurred to hospitals [18]. Unfortunately, accurate estimates of cost-to-charge ratios for specific healthcare services provided by hospitals in China are not currently available. To address this issue, the average cost-to-charge ratio (0.925) of the study hospital during 2017–2018 was obtained from its annual financial reports. Direct medical expenditures were converted into opportunity costs of care to the study hospital using this ratio.

From the responses provided by patients who completed the inpatient module during the telephone survey, costs per inpatient episode were calculated by adding up the direct medical costs, the direct non-medical costs, and the indirect costs. Generalized linear regression models (GLM) were used to identify predictors of costs. Demographic, socioeconomic, and clinical characteristics and treatment methods were investigated.

Then, total costs per case by clinical stages from diagnosis to one year after final discharge were extrapolated. The treatment processes were broadly divided into two phases: the inpatient phase (from diagnosis to final discharge) and the outpatient phase (one year after final discharge).

For the inpatient phase, direct medical costs were calculated based on two parameters: first, the average direct medical costs associated with inpatient episodes, which were estimated by treatment type using all eligible inpatient records; and second, the average number of hospitalization, which was estimated by treatment type received during the entire inpatient phase using only records of patients who experienced final discharge. Using the group of patients with IA cervical cancer as an example: there were 37 patients who experienced final discharge, 36 of whom were hospitalized once for surgery, and one patient for five times (once for surgery, and the other four for chemotherapy). Therefore, the average number of hospitalization with surgery and chemotherapy received per IA patient was 1 (37/37) and 0.11 (4/37), respectively. Direct medical costs were calculated by multiplying the aforementioned parameters for each treatment type and then adding up their products for each clinical stage. Direct non-medical costs and indirect costs were calculated by multiplying the average of the two costs associated with each inpatient episode with the average number of hospitalizations during the inpatient phase. If the respondents provided an un-useable answer to indirect costs, their corresponding estimates were derived using the human capital method based on national average income per capita and their losses of workdays.

In the outpatient phase, the Guidelines for Diagnosis and Treatment of Cervical Cancer, issued by the Committee of Gynecological Oncology of the Chinese Anti-Cancer Association, recommends patients to be regularly followed-up as outpatients every two months in the first six-month period and every three months in the second six-month period after final discharge, totaling five outpatient visits [19]. Thus, total costs during the outpatient phase were obtained by multiplying the sum of the average direct medical and non-medical costs associated with each outpatient visit by five.

To examine the quality of life impact of cervical cancer, responses to the EQ-5D descriptive system for the current and worst health states were converted to health utilities using a China-specific value set [20]. The baseline utility for patients at diagnosis was assumed to be the norms for the Chinese population. The worst health states were expected to occur right after the first inpatient treatments. The loss of QALY associated with cervical cancer from diagnosis to the interview day was obtained by comparing different utilities at diagnosis, the worst health state, and the interview using the ‘under the curve’ method. Linear changes in EQ-5D scores over time from diagnosis to the worst health state and from the worst health state to the interview were assumed. The total QALY loss from diagnosis to one year after final discharge was predicted based on results from the GLMs while controlling for time since diagnosis and the full list of patient characteristics. Eventually, the average QALY loss per case by clinical stages was obtained by averaging the corresponding predicted values.

In this study, the patient information was stored in coded encryption after data collection, and we had no access to information that could identify individual participants. We adjusted for inflation to match for 2018 US dollar value using a country-specific consumer price index and foreign exchange rates [21]. All statistical analyses were performed using R version 3.6.1 for Windows.

### 2.4. Ethical considerations

The study was reviewed and approved by the institutional review board at Henan Cancer Hospital (approval number: 2019010) and Fudan University School of Public Health (approval number: IRB#2018-10-0710).

## 3. Results

### 3.1. Patient characteristics

From January 2017 to December 2018, there were a total of 4,169 inpatient admissions to the Gynecology Department at the study hospital. Of these, 3,506 records of inpatient episodes from 1,323 patients were eligible under the inclusion criteria. Among them, 844 patients had experienced final discharge and 541 (40%) randomly selected patients were invited to the telephone survey. Module completion rates varied from 26.1% (141/541) (the module on direct costs for outpatient visits) to 61.0% (330/541) (the EQ-5D module). Main reasons for non-completion included unwillingness to provide information, language barriers, and distrust of investigators.

Table 1 describes patient characteristics. Overall, the majority of the patients were aged above 45 years at diagnosis, living in rural areas, unemployed, and confirmed with less advanced cervical cancer (IA-IIA). Generally, there were no significant differences between patients who are involved in the analysis and those who are not. However, patients who responded to the EQ-5D module had less advanced cancer compared to those who did not (col. (6) of S1 Table). Stronger unwillingness to participate in the survey rather than higher mortality among more advanced patients was responsible for this difference (S1 and S2 Tables in the supplementary materials).

**Table 1:** Demographics, socioeconomic status, and clinical characteristics of patients involved in various stages of the study (n, %).

	Patients involved in the analysis who…					
	were admitted to the study hospital	experienced final discharges	responded to inpatient questions	provided direct medical costs for outpatient visits	provided direct non-medical costs for outpatient visits	responded to EQ-5D questions
	(1)	(2)	(3)	(4)	(5)	(6)
Total	1323 (100)	844 (100)	225 (100)	141 (100)	158 (100)	330 (100)
***Demographics***						
Age at diagnosis						
<45	335 (25.3)	225 (26.7)	61 (27.1)	41 (29.1)	40 (25.3)	89 (27.0)
> = 45	988 (74.7)	619 (73.3)	164 (72.9)	100 (70.9)	118 (74.7)	241 (73.0)
Area of residence						
Urban	334 (25.2)	219 (25.9)	52 (23.1)	33 (23.4)	39 (24.7)	85 (25.8)
Rural	989 (74.8)	625 (74.1)	173 (76.9)	108 (76.6)	119 (75.3)	245 (74.2)
Marital status						
Married	1296 (98.0)	827 (98.0)	218 (96.9)	135 (95.7)	150 (94.9)	321 (97.3)
Unmarried	27 (2.0)	17 (2.0)	7 (3.1)	6 (4.3)	8 (5.1)	9 (2.7)
***Socioeconomic status***						
Education level						
Elementary			74 (32.9)	51 (36.2)	60 (38.0)	99 (30.0)
Junior school			84 (37.3)	51 (36.2)	53 (33.5)	119 (36.1)
High school			50 (22.2)	29 (20.5)	33 (20.9)	62 (18.8)
College			15 (6.7)	10 (7.1)	11 (7.0)	22 (6.7)
Unknown			2 (0.9)	0 (0)	1 (0.6)	28 (8.5)
Monthly family Income (RMB)						
< 5K			132 (58.7)	86 (61.0)	99 (62.7)	165 (50.0)
5K-10K			28 (12.4)	18 (12.8)	18 (11.4)	43 (13.0)
> 10K			17 (7.6)	10 (7.1)	11 (7.0)	24 (7.3)
Unknown			48 (21.3)	27 (19.1)	30 (18.9)	98 (29.7)
Insurance type						
URBMI	36 (2.7)	21 (2.5)	7 (3.1)	2 (1.4)	3 (1.9)	11 (3.3)
UEBMI	139 (10.5)	92 (10.9)	25 (11.1)	18 (12.8)	18 (11.4)	33 (10.0)
NCMS	851 (64.3)	533 (63.2)	152 (67.6)	95 (67.4)	107 (67.7)	219 (66.4)
Others	58 (4.4)	36 (4.3)	6 (2.7)	4 (2.8)	7 (4.4)	13 (3.9)
No insurance	239 (18.1)	162 (19.2)	35 (15.5)	22 (15.6)	23 (14.6)	54 (16.4)
Employment status						
Employed	496 (37.5)	315 (37.3)	79 (35.1)	50 (35.5)	56 (35.4)	116 (35.2)
Unemployed	827 (62.5)	529 (62.7)	146 (64.9)	91 (64.5)	102 (64.6)	214 (64.9)
***Clinical characteristics***						
Clinical stage						
IA	42 (3.2)	37 (4.4))	11 (4.9)	8 (5.7)	7 (4.4)	15 (4.6)
IB	585 (44.2)	402 (47.6)	103 (45.7)	65 (46.0)	72 (45.5)	167 (50.6)
IIA	389 (29.4)	203 (24.1)	71 (31.6)	39 (27.6)	47 (29.7)	88 (26.7)
IIB	134 (10.1)	95 (11.3)	22 (9.8)	12 (8.5)	14 (8.9)	27 (8.2)
III	135 (10.2)	93 (11.0)	16 (7.1)	17 (12.0)	18 (11.3)	30 (9.1)
IV	38 (2.9)	14 (1.7)	2 (0.9)	0 (0.0)	0 (0.0)	3 (0.9)
Pathological type						
Squamous cell	1113 (84.1)	734 (84.3)	199 (88.4)	125 (88.7)	140 (88.6)	279 (84.6)
Adenocarcinoma	127 (9.6)	84 (9.6)	15 (6.7)	9 (6.4)	9 (5.7)	33 (10.0)
Other	26 (2.0)	16 (1.8)	4 (1.8)	3 (2.1)	3 (1.9)	5 (1.5)
Unknown	57 (4.3)	37 (4.2)	7 (3.1)	4 (2.8)	6 (3.8)	13 (3.9)

The table presents patient counts and percentages out of total for each group demographic and clinical characteristics across all analytical steps. URBMI stands for Urban Resident Basic Medical Insurance, UEBMI for Urban Employee Basic Medical Insurance, and NCMS for New Cooperative Medical Scheme.

The distribution of inpatient phase duration among those who experienced final discharge in our study shows that patients with advanced cancers generally underwent more extended inpatient phases (see S1 Fig). For IA patients, their inpatient phase duration was around 23 days. In contrast, IB-III patients spent around 100 days and IV patients spent on average 147 days due to more rounds of chemotherapy.

### 3.2. Costs associated with cervical cancer per inpatient episode

For costs per inpatient episode, clinical stage and treatment methods were significantly associated with total costs, direct medical costs, and direct non-medical costs (col. (1)-(3) of Table 2). Rural residence was also a strong predictor of higher direct non-medical costs (col. (3) of Table 2). By contrast, older age was associated with lower indirect costs and those employed paid higher indirect costs (col. (4) of Table 2).

**Table 2:** Statistically significant predictors of costs per inpatient episode (US$) (coefficients, confidence interval).

	Costs per inpatient episode			
	Total	Direct medical	Direct non-medical	Indirect

	(1)	(2)	(3)	(4)
Age at first diagnosis (reference group: < 45)				
> = 45	─	─	─	-2445.5***
	─	─	─	(-3359.1, -1531.9)
Area of residence (reference group: urban)				
Rural	─	─	1579.7***	─
	─	─	(957.2, 2202.1)	─
Employment status (reference group: unemployed)				
Employed	─	─		979.1*
	─	─		(118.0, 1840.1)
Clinical stage (reference group: IA)				
IB	5970	4382.0	1355.9	─
	(-801.6, 12741.5)	(-939.5, 9703.5)	(-442.1, 3153.8)	─
IIA	6538	6017.3*	1020.3	─
	(-308.0, 13383.1)	(637.6, 11396.9)	(-803.8, 2844.4)	─
IIB	13708**	14304.5***	3761.4**	─
	(5155.4, 22260.6)	(7583.4, 21025.6)	(1490.8, 6032.0)	─
III	25060***	22691.9***	3212.7**	─
	(16661.1, 33457.9)	(16091.9, 29291.8)	(983.1, 5442.3)	─
IV	7584	7530.0	724.1	─
	(-3019.4, 18187.8)	(-803.0, 15862.9)	(-2095.1, 3543.3)	─
Treatment methods (reference group: surgery)				
Chemotherapy	-25883***	-22827.5***	-867.2**	-2578.3***
	(-28114.8, -23651.5)	(-24581.3, -21073.7)	(-1460.3, -274.2)	(-3520.8, -1635.9)
Radiotherapy	30507***	27014.5***	-1460	3907.3**
	(23765.1, 37248.3)	(21716.6, 32312.5)	(-3249.7, 329.6)	(1074.9, 6739.7)
Surgery and chemo combo	11754**	7100.6*	248.2	4520.8**
	(4670.0 18808.8)	1556.9, 12644.4)	(-1628.2, 2124.5)	(1439.0, 7602.5)
Radio and chemo combo	47640***	35564.1***	1887.4***	9363.0***
	(44222.1, 51058.2)	(32878.0, 38250.2)	(979.7, 2795.1)	(8019.1, 10706.8)
Surgery, radio and chemo combo	77726***	72625.3***	-3146.3	2506.2
	(61554.7, 93896.5)	(59917.2, 85333.4)	(-7440.6, 1148.0)	(-4215.2, 9227.6)
Sample size	575	575	575	575

Results were produced from participants who successfully answered questions regarding inpatient episodes. For each costs category, patient characteristics with p-value above 0.10 in univariate analyses were excluded and the final model specification was selected according to minimum Akaike information criterion (AIC) stepwise regression procedure.

* for significant at the 5% level

** for significant at the 1% level

*** for significant at the 0.1% level.

**Fig 2:**
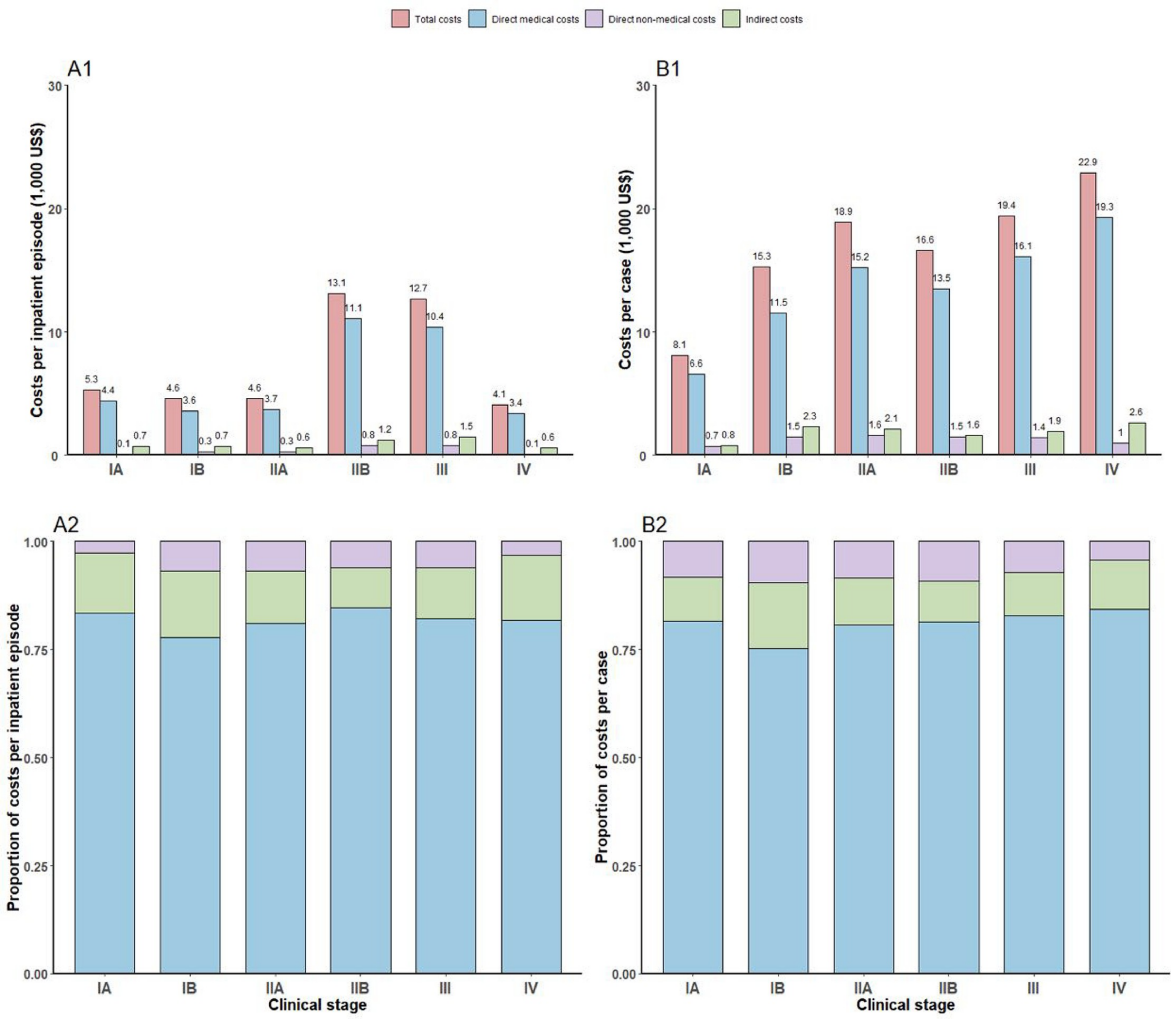
Costs per inpatient episodes and costs per case from diagnosis to final discharge, by clinical stages. Costs per inpatient episode were described in panel A1. The proportion of costs per inpatient episode by components was presented in panel A2. Costs per case from diagnosis to final discharge were described in panel B1. The proportion of costs per inpatient episode by components was presented in panel B2. Values presented in the figure were rounded to one digit decimal place.

Fig 2 presents costs per inpatient episode (Panel A1) and the proportion of total costs by components (Panel A2) across clinical stages. On average, total costs per inpatient episode were $5,321, $4,589, $4,586, $13,084, $12,663, $4,143 for IA-IV patients, respectively. Compared to patients of the other clinical stages, IIB and III patients spent almost two times more. Across all clinical stages, direct medical costs accounted for the largest shares of total costs (77.7%-84.7%), followed by indirect costs (9.2%-15.3%).

### 3.3. Costs per case from diagnosis to one year after discharge

Table 3 presents costs per case from diagnosis to one year after final discharge by clinical stages. Direct medical costs during the inpatient phase and indirect costs increased with clinical stages and were the top and the second largest components of total costs, with the exception of IIB patients (Panel B2 of Fig 2). For IIB patients, indirect costs were slightly lower than direct non-medical costs. Unfortunately, we were not able to obtain estimates on direct medical and non-medical costs for the outpatient phase for IV patients (col. (2) and (4) of Table 3). Only a small number of IV patients were admitted and none of the IV patients who we invited for the telephone interview completed the modules regarding direct medical and non-medical costs associated with outpatient visits. Since estimates on the direct medical costs and direct non-medical costs during the outpatient phase did not vary much across clinical stages, we assumed that the values for IV patients were the same as those for III patients. On average, total costs per cervical cancer patient from diagnosis to one year after final discharge were $8,066, $15,260, $18,873, $16,630, $19,440, $22,888 for IA-IV patients, respectively.

**Table 3:** Costs and loss in quality-adjusted life years per case from diagnosis to one year after final discharge, by clinical stages (US$).

	Direct medical costs		Direct non-medical costs		Indirect costs	Total costs	QALY loss
	Inpatient phase	Outpatient phase	Inpatient phase	Outpatient phase			
	(1)	(2)	(3)	(4)	(5)	(6)	(7)
Clinical stage							
IA	4747	1832	163	510	814	8066	0.05
IB	9827	1649	1050	409	2325	15260	0.17
IIA	13799	1405	1152	437	2080	18873	0.23
IIB	12263	1272	1049	487	1559	16630	0.21
III	14417	1669	1014	408	1933	19440	0.26
IV*	17617	1669	571	408	2623	22888	0.19

*Values for III patients were used for columns (2) and (4). Total costs in column (6) were calculated accordingly.

### 3.4. QALY loss per case

The EQ-5D scores of the descriptive system and VAS suggest similar patterns. Compared to the norms of the Chinese population, cervical cancer patients had poorer health states (S3 Table). The average total QALY loss for a cervical cancer patient from diagnosis to one year after final discharge was 0.054, 0.167, 0.230, 0.205, 0.257 and 0.190 for clinical stages of IA-IV, respectively (col. (7) of Table 3).

## 4. Discussion

### 4.1. Principal findings

This study adopted a societal perspective and examined clinical stage specific costs and QALY loss associated with cervical cancer from diagnosis to one year after final discharge in Henan Province, China. Based on the inpatient records from the largest cancer hospital in the province and a retrospective telephone survey, we found that costs per case ranged from $8,066-$22,888 and QALY loss ranged from 0.05–0.26.

To further understand the financial burden, we calculated the average time from diagnosis to one year after final discharge by clinical stages. We then divided total costs per case by these time estimates. The overall average was weighted by the proportion of each clinical stage admitted to the study hospital. We found that the average annual costs per cervical cancer patient were 1.7 times GDP per capita and 4.0 times per capita household disposable income in Henan Province as of 2018. According to a recent systematic review, patients in middle- and high-income countries only spend 75.52% and 30.12% of the local GDP per capita on cervical cancer treatment [22]. Our results suggest that from the societal perspective, the economic burden of cervical cancer in Henan province is much higher than these countries.

### 4.2. Comparison with other studies

Previous studies from China mainly focused on total direct medical expenditures during the inpatient phase [23] or the average medical expenditures associated with inpatient episodes [24–28]. To support comparison with existing studies, we also produced these results. Based on a national sample of cervical cancer patients, Tao et al. estimated that the direct economic burden associated with hospitalization in Chinese cities was $7,706 for patients at early stages (IA-IIA) and $13,319 for patients at advanced stages (IIB-IV) (results adjusted to US dollar in 2018) [23]. In contrast, our study produced larger estimates mainly because our calculation was based on patients who have completed the entire inpatient phase of their treatment process rather than all patients admitted to hospitals during a given period. We noted that costs estimates for patients could be inaccurate if the entire treatment process was not fully recognized.

Although previous studies that reported expenditures per inpatient episode were conducted in different areas of China [24–28], we found similar results that clinical stages and treatment methods were important predictors of direct medical costs associated with hospitalization (col. (2) of Table 2).Moreover, we found that direct non-medical costs of hospitalization were higher among rural residents due to larger travel costs (col. (3) of Table 2). Indirect costs were higher among the employed due to higher income loss (col. (4) of Table 2).

Our study contributed to the existing literature on the economic burden of cervical cancer in China due to a number of features. First, our estimates suffer less under-estimation because we accounted for a wide range of costs categories and considered the entire inpatient phase and a one-year outpatient phase. We highlighted the importance of indirect costs and costs during the outpatient phase as a component of financial burden, which makes up 9.4%-15.2% and 9.1%-29.0% of total costs (Table 3, Panel B2 of Fig 2). Second, we provided more detailed estimates for each clinical stage rather than broadly categorized patients into early stages and advanced stages. Third, we estimated QALY loss from diagnosis to one year after final discharge, which is an important parameter for cost-effectiveness analyses on interventions against cervical cancer. Fourth, our study was based on a large sample of cervical cancer patients and inpatient records, which added to the accuracy of our results.

### 4.3. Policy implications

This study speaks to the important policy concern on whether it is cost-effective for the central government of China to add the HPV vaccine into its national immunization program. Currently, HPV vaccines in the China market are produced by two foreign pharmaceuticals (GlaxoSmithKline and Merk & Co.) and injection costs are fully borne by individuals. Although the average annual costs per cervical patient were estimated to be 4.0 times per capita household disposable income in Henan Province, the costs of three doses of HPV vaccine are also expensive to afford (3.3%-10.4% of per capita household disposable income). In January 2020, a newly approved domestic bivalent HPV vaccine is being prepared for production. It is priced at nearly half of the costs of those provided by foreign drugmakers, which potentially increases the cost-effectiveness of the injection.

Unfortunately, previous study on the economic burden of cervical cancer in China did not fully recognize the long and complicated treatment process of the disease, leading to an underestimation of the costs incurred to the whole society that HPV vaccines can prevent. Our study instead produced more accurate estimates for the treatment costs of cervical cancer and loss in quality-adjusted life years associated with the disease. These results provided crucial parameters for cost-effectiveness studies and are important for the decision of relevant immunization policy.

### 4.4. Limitations

Our study is subject to several limitations. First, information collected from the telephone surveys may be subject to recall bias. Information on non-medical and indirect costs may suffer measurement errors since it was obtained based on the memory of the participants. Second, the response rate of the telephone survey was not ideal which may potentially affect the accuracy of our results. Comparisons between patients in the final analysis and those not (S1 and S2 Tables) suggested no overall significant differences in their characteristics. However, results for IV patients might be less accurate due to the limited number of patients who provided answers during the survey. Third, we used the norms as the health states for patients at diagnosis, which, however, could lead to over-estimation. And we assumed that the worst health states occurred on the day of the first treatment, which may be less valid for patients who did not make a good recovery. Fourth, due to lack of data, we did not consider impacts of early mortality associated with cervical cancer in our analysis. Impacts of long-term consequences could be a direction of future studies. Fifth, the disease burden of cervical cancer varies substantially across areas with different levels of development in China. To provide more informative parameters for cost-effectiveness analysis, area-specific costs estimates are also important, which could be the focus of future studies.

## Supporting information

S1 Table Comparison of characteristics among patients involved in various stages of the study (n (%), p-value). (DOCX)

S2 Table Comparison of characteristics between patients successfully interviewed and those not (n (%), p-value). (DOCX)

S3 Table EQ-5D scores of the descriptive system and visual analogue scale (VAS), by clinical stages (mean, standard deviation). (DOCX)

S1 Fig Distribution of inpatient phase duration among patients experienced final discharges, by clinical stages. (TIF)

S1 Questionnaires (ZIP)

S1 File (ZIP)

## Abbreviations

HPV: Human papillomavirus
LMIC: low- and middle-income countries
QALY: quality-adjusted life years
VAS: visual analogue scale
GLM: generalized linear regression models
