# Supplementary material for: The economic burden of cervical cancer from diagnosis to one year after final discharge in Henan Province, China: A retrospective case series study
Source: PLoS One. 2020 May 7;15(5):e0232129. doi: 10.1371/journal.pone.0232129 (PMC7205285; doi:10.1371/journal.pone.0232129)
Supplement: S1 Table — (DOCX) [file pone.0232129.s001.docx]

Table S1. Comparison of characteristics among patients involved in various stages of the study (n (%), p-value)

|  | Patients invited for telephone interview | P-value (versus col. (1) of Table 1) |  | Patients  did not experience final discharged | P-value (versus col. (2) of Table 1) |  | Interview invited but inpatient questions not answered | P-value (versus col. (3) of Table 1) |
| --- | --- | --- | --- | --- | --- | --- | --- | --- |
|  | (1) | (2) |  | (3) | (4) |  | (5) | (6) |
|  |  |  |  |  |  |  |  |  |
| Total | 541 (100) |  |  | 479 (100) |  |  | 316 (100) |  |
| Age at diagnosis |  | 0.25 |  |  | 0.14 |  |  | 0.73 |
| <45 | 151 (27.9) |  |  | 110 (23.0) |  |  | 90 (28.5) |  |
| >=45 | 390 (72.1) |  |  | 369 (77.0) |  |  | 226 (71.5) |  |
| Area of residence |  | 0.91 |  |  | 0.44 |  |  | 0.28 |
| Urban | 138 (25.5) |  |  | 115 (24.0) |  |  | 86 (27.2) |  |
| Rural | 403 (74.5) |  |  | 364 (76.0) |  |  | 230 (72.8) |  |
| Marital status |  | 0.33 |  |  | 0.93 |  |  | 0.69 |
| Married | 526 (97.2) |  |  | 469 (97.9) |  |  | 308 (97.5) |  |
| Unmarried | 15 (2.8) |  |  | 10 (2.1) |  |  | 8 (2.5) |  |
| Education level |  |  |  |  |  |  |  | 0.60 |
| Elementary | ─ |  |  | ─ |  |  | 41 (13.0) |  |
| Junior school | ─ |  |  | ─ |  |  | 44 (13.9) |  |
| High school | ─ |  |  | ─ |  |  | 20 (6.3) |  |
| College | ─ |  |  | ─ |  |  | 11 (3.5) |  |
| Unknown | ─ |  |  | ─ |  |  | 200 (63.3) |  |
| Monthly family  Income (RMB) |  |  |  |  |  |  |  | 0.14 |
| < 5K | ─ |  |  | ─ |  |  | 49 (15.5) |  |
| 5K-10K | ─ |  |  | ─ |  |  | 20 (6.3) |  |
| > 10K | ─ |  |  | ─ |  |  | 8 (2.5) |  |
| Unknown | ─ |  |  | ─ |  |  | 239 (75.7) |  |
| Insurance type |  | 0.76 |  |  | 0.56 |  |  | 0.80 |
| URBMI | 16 (3.0) |  |  | 15 (3.1) |  |  | 9 (2.8) |  |
| UREMI | 55 (10.2) |  |  | 47 (9.8) |  |  | 30 (9.5) |  |
| NCMS | 358 (66.2) |  |  | 318 (66.4) |  |  | 206 (65.2) |  |
| Others | 17 (3.1) |  |  | 22 (4.6) |  |  | 11 (3.5) |  |
| No insurance | 95 (17.6) |  |  | 77 (16.1) |  |  | 60 (19.0) |  |
| Employment status |  | 0.95 |  |  | 0.87 |  |  | 0.37 |
| Employed | 202 (37.3) |  |  | 181 (37.8) |  |  | 123 (38.9) |  |
| Unemployed | 339 (62.7) |  |  | 298 (62.2) |  |  | 193 (61.1) |  |
| Clinical stage (FIGO) |  | 0.47 |  |  | 0.40 |  |  | 0.06 |
| IA-IIA | 423 (78.2) |  |  | 374 (78.1) |  |  | 238 (75.3) |  |
| IIB-IV | 118 (21.8) |  |  | 105 (21.9) |  |  | 78 (24.7) |  |
| Pathological type |  | 0.95 |  |  | 0.88 |  |  | 0.20 |
| Squamous cell | 459 (84.8) |  |  | 404 (84.3) |  |  | 260 (82.3) |  |
| Adenocarcinoma | 52 (9.6) |  |  | 45 (9.4) |  |  | 37 (11.7) |  |
| Others | 9 (1.7) |  |  | 11 (2.3) |  |  | 5 (1.6) |  |
| Unknown | 21 (3.9) |  |  | 19 (4.0) |  |  | 14 (4.4) |  |

Notes: The table presents counts and percentages of total patients in parentheses for each group of patient characteristics in odd columns and p-value of chi-square tests in even columns. URBMI stands for Urban Resident Basic Medical Insurance, UEBMI for Urban Employee Basic Medical Insurance, and NCMS for New Cooperative Medical Scheme.
