# Supplementary material for: The economic burden of cervical cancer from diagnosis to one year after final discharge in Henan Province, China: A retrospective case series study
Source: PLoS One. 2020 May 7;15(5):e0232129. doi: 10.1371/journal.pone.0232129 (PMC7205285; doi:10.1371/journal.pone.0232129)
Supplement: S2 Table — (DOCX) [file pone.0232129.s002.docx]

Table S2. Comparison of characteristics between patients successfully interviewed and those not (n (%), p-value)

|  | Interview invited but no direct medical costs for outpatient visits collected | P-value (versus col. (4) of Table 1) |  | Interview invited but no direct non-medical costs for outpatient visits collected | P-value (versus col. (5) of Table 1) |  | Interview invited but  no EQ-5D answers collected | P-value (versus  col. (6) of Table 1) |
| --- | --- | --- | --- | --- | --- | --- | --- | --- |
|  | (1) | (2) |  | (3) | (4) |  | (5) | (6) |
|  |  |  |  |  |  |  |  |  |
| Total | 400 (100) |  |  | 383 (100) |  |  | 211 (100) |  |
| Age at diagnosis |  | 0.72 |  |  | 0.39 |  |  | 0.39 |
| <45 | 110 (27.5) |  |  | 111 (29.0) |  |  | 62 (29.4) |  |
| >=45 | 290 (72.5) |  |  | 272 (71.0) |  |  | 149 (70.6) |  |
| Area of residence |  | 0.51 |  |  | 0.78 |  |  | 0.78 |
| Urban | 105 (26.3) |  |  | 99 (25.8) |  |  | 53 (25.1) |  |
| Rural | 295 (73.7) |  |  | 284 (74.2) |  |  | 158 (74.9) |  |
| Marital status |  | 0.21 |  |  | 0.04 |  |  | 0.04 |
| Married | 391 (97.7) |  |  | 376 (98.2) |  |  | 205 (97.2) |  |
| Unmarried | 9 (2.3) |  |  | 7 (1.8) |  |  | 6 (2.8) |  |
| Education level |  | 0.89 |  |  | 0.39 |  |  | 0.39 |
| Elementary | 64 (16.0) |  |  | 55 (14.4) |  |  | 16 (7.6) |  |
| Junior school | 77 (19.3) |  |  | 75 (19.6) |  |  | 9 (4.3) |  |
| High school | 41 (10.2) |  |  | 37 (9.7) |  |  | 8 (3.8) |  |
| College | 16 (4.0) |  |  | 15 (3.9) |  |  | 4 (1.9) |  |
| Unknown | 202 (50.5) |  |  | 201 (52.5) |  |  | 174 (82.5) |  |
| Monthly family  Income (RMB) |  | 0.41 |  |  | 0.08 |  |  | 0.08 |
| < 5K | 95 (23.7) |  |  | 82 (21.4) |  |  | 16 (7.6) |  |
| 5K-10K | 30 (7.5) |  |  | 30 (7.8) |  |  | 5 (2.4) |  |
| > 10K | 15 (3.8) |  |  | 14 (3.7) |  |  | 1 (0.5) |  |
| Unknown | 260 (65.0) |  |  | 257 (67.1) |  |  | 189 (89.6) |  |
| Insurance type |  | 0.51 |  |  | 0.47 |  |  | 0.47 |
| URBMI | 14 (3.5) |  |  | 13 (3.4) |  |  | 5 (2.4) |  |
| UREMI | 37 (9.3) |  |  | 37 (9.7) |  |  | 22 (10.4) |  |
| NCMS | 263 (65.7) |  |  | 251 (65.5) |  |  | 139 (65.9) |  |
| Others | 13 (3.3) |  |  | 10 (2.6) |  |  | 4 (1.9) |  |
| No insurance | 73 (18.2) |  |  | 72 (18.8) |  |  | 41 (19.4) |  |
| Employment status |  | 0.59 |  |  | 0.56 |  |  | 0.56 |
| Employed | 152 (38.0) |  |  | 146 (38.1) |  |  | 86 (40.8) |  |
| Unemployed | 248 (62.0) |  |  | 237 (61.9) |  |  | 125 (59.2) |  |
| Clinical stage (FIGO) |  | 0.68 |  |  | 0.57 |  |  | 0.57 |
| IA-IIA | 311 (77.8) |  |  | 297 (77.5) |  |  | 153 (72.5) |  |
| IIB-IV | 89 (22.3) |  |  | 86 (22.5) |  |  | 58 (27.5) |  |
| Pathological type |  | 0.37 |  |  | 0.26 |  |  | 0.26 |
| Squamous cell | 334 (83.5) |  |  | 319 (83.3) |  |  | 180 (85.3) |  |
| Adenocarcinoma | 43 (10.7) |  |  | 43 (11.2) |  |  | 19 (9.0) |  |
| Others | 6 (1.5) |  |  | 6 (1.6) |  |  | 4 (1.9) |  |
| Unknown | 17 (4.3) |  |  | 15 (3.9) |  |  | 8 (3.8) |  |

Notes: The table presents counts and percentages of total patients in parentheses for each group of patient characteristics in odd columns and p-value of chi-square tests in even columns. URBMI stands for Urban Resident Basic Medical Insurance, UEBMI for Urban Employee Basic Medical Insurance, and NCMS for New Cooperative Medical Scheme.
