# Supplementary material for: The economic burden of cervical cancer from diagnosis to one year after final discharge in Henan Province, China: A retrospective case series study
Source: PLoS One. 2020 May 7;15(5):e0232129. doi: 10.1371/journal.pone.0232129 (PMC7205285; doi:10.1371/journal.pone.0232129)
Supplement: S3 Table — (DOCX) [file pone.0232129.s003.docx]

Table S3. EQ-5D scores of the descriptive system and visual analogue scale (VAS), by clinical stages (mean, standard deviation)

|  | EQ-5D-5L | | |  | VAS | | |
| --- | --- | --- | --- | --- | --- | --- | --- |
|  | Norms | Worst  state | At  interview |  | Norms | Worst  state | At  interview |
|  | (1) | (2) | (3) |  | (4) | (5) | (6) |
|  |  |  |  |  |  |  |  |
| Clinical stages |  |  |  |  |  |  |  |
| IA | 0.96 | 0.86 | 0.92 |  | 86.60 | 54.90 | 68.80 |
|  |  | (0.12) | (0.04) |  |  | (16.80) | (7.50) |
| IB | 0.96 | 0.76 | 0.90 |  | 86.60 | 48.40 | 72.50 |
|  |  | (0.32) | (0.16) |  |  | (18.80) | (14.00) |
| IIA | 0.96 | 0.65 | 0.89 |  | 86.60 | 54.90 | 70.30 |
|  |  | (0.34) | (0.11) |  |  | (13.00) | (11.90) |
| IIB | 0.96 | 0.74 | 0.92 |  | 86.60 | 56.30 | 74.00 |
|  |  | (0.31) | (0.07) |  |  | (11.10) | (8.70) |
| III | 0.96 | 0.63 | 0.86 |  | 86.60 | 43.10 | 71.60 |
|  |  | (0.36) | (0.19) |  |  | (22.40) | (15.60) |
| IV | 0.96 | 0.71 | 0.73 |  | 86.60 | 51.60 | 51.60 |
|  |  | (0.34) | (0.35) |  |  | -- | -- |

Notes: Results in columns (2) and (3) were produced from the sample of participants who were invited to the telephone interview and successfully answered the descriptive part of the EQ-5D questions. Results in columns (5)-(6) were produced from participants who also answered the VAS questions. The sample sizes in columns (5) and (6) were slightly smaller compared to those in columns (2) and (3) Standard deviations were not obtained for IV patients in columns (5) and (6) because only one IV patient provided answers to the VAS question.
