# Supplementary figures and images for: The economic burden of cervical cancer from diagnosis to one year after final discharge in Henan Province, China: A retrospective case series study
Source: PLoS One. 2020 May 7;15(5):e0232129. doi: 10.1371/journal.pone.0232129 (PMC7205285; doi:10.1371/journal.pone.0232129)

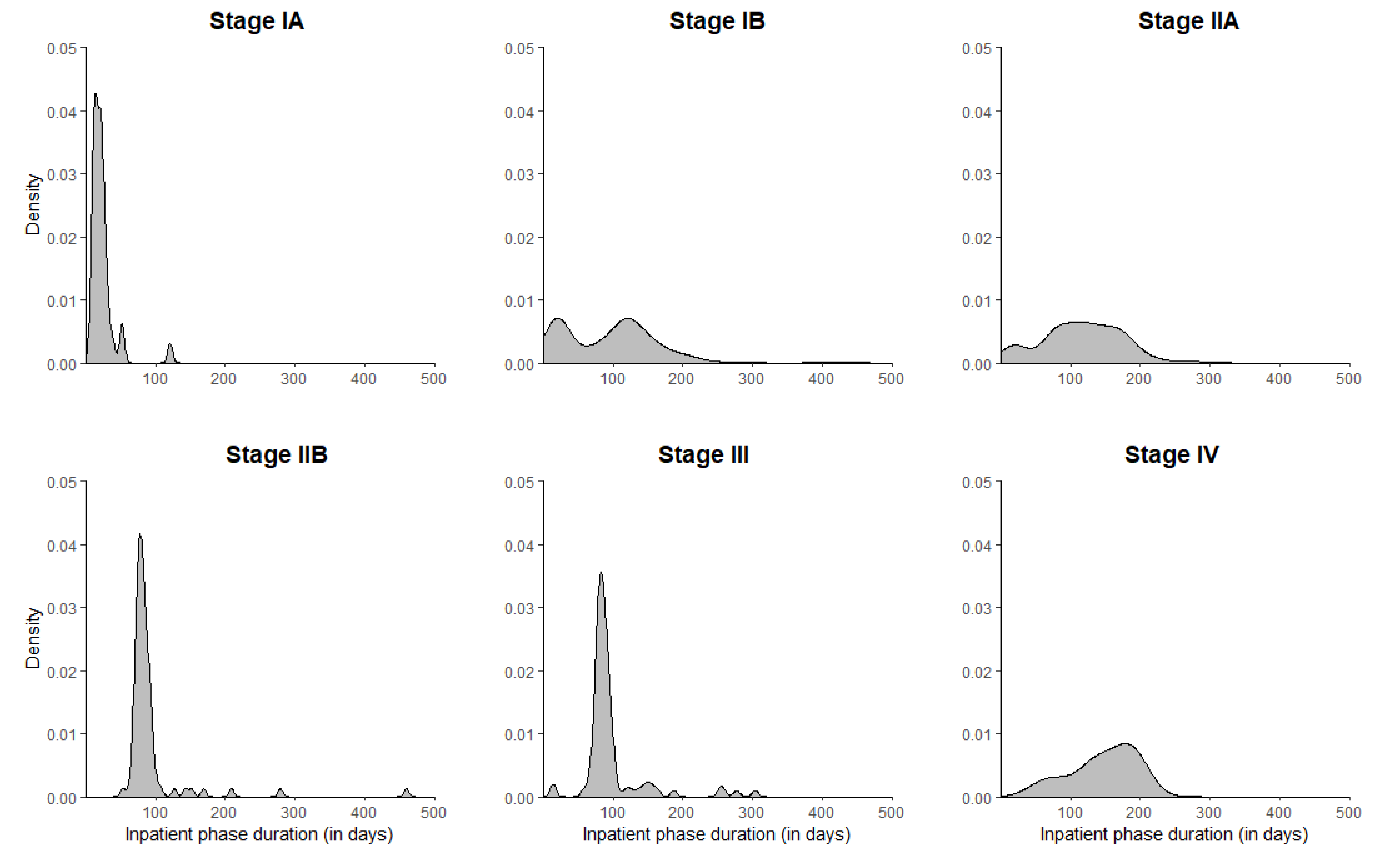

Supplement: S1 Fig — (TIF) [file pone.0232129.s004.tif]
