# Supplementary material for: The economic burden of cervical cancer from diagnosis to one year after final discharge in Henan Province, China: A retrospective case series study
Source: PLoS One. 2020 May 7;15(5):e0232129. doi: 10.1371/journal.pone.0232129 (PMC7205285; doi:10.1371/journal.pone.0232129)
Supplement: S1 Questionnaires — (ZIP) [file pone.0232129.s005.zip › Questionnaires/From HIS_English.docx]

**Information from HIS**

| No. | Variable | Text |
| --- | --- | --- |
| Q1 | Patient ID |  |
| Q2 | Name |  |
| Q3 | InPatient No. |  |
| Q4 | Contact information |  |
| Q5 | Date of birth |  |
| Q6 | Date of first diagnosis |  |
| Q7 | Number of admission |  |
| Q8 | Date of admission |  |
| Q9 | Date of discharge |  |
| Q10 | Discharge diagnosis |  |
| Q11 | Clinical stage |  |
| Q12 | Pathological type |  |
| Q13 | Surgery type |  |
| Q14 | Medicine charge |  |
| Q15 | Surgery charge |  |
| Q16 | Radiotherapy charge |  |
| Q17 | Total hospitalization charge |  |
