# Supplementary material for: The economic burden of cervical cancer from diagnosis to one year after final discharge in Henan Province, China: A retrospective case series study
Source: PLoS One. 2020 May 7;15(5):e0232129. doi: 10.1371/journal.pone.0232129 (PMC7205285; doi:10.1371/journal.pone.0232129)
Supplement: S1 Questionnaires — (ZIP) [file pone.0232129.s005.zip › Questionnaires/From HIS_origin.docx]

**患者信息**

| 序号 | 变量 | 内容 |
| --- | --- | --- |
| Q1 | 患者ID |  |
| Q2 | 患者姓名 |  |
| Q3 | 患者住院号 |  |
| Q4 | 联系方式 |  |
| Q5 | 出生日期 |  |
| Q6 | 首次诊断日期 |  |
| Q7 | 入院次数 |  |
| Q8 | 入院日期 |  |
| Q9 | 出院日期 |  |
| Q10 | 出院诊断 |  |
| Q11 | 临床分期 |  |
| Q12 | 病理类型 |  |
| Q13 | 手术名称 |  |
| Q14 | 西药费 |  |
| Q15 | 手术费 |  |
| Q16 | 放疗费 |  |
| Q17 | 住院总费用 |  |
