# Supplementary material for: The economic burden of cervical cancer from diagnosis to one year after final discharge in Henan Province, China: A retrospective case series study
Source: PLoS One. 2020 May 7;15(5):e0232129. doi: 10.1371/journal.pone.0232129 (PMC7205285; doi:10.1371/journal.pone.0232129)
Supplement: S1 File — (ZIP) [file pone.0232129.s006.zip › Ethics Approvals/Ethics approval_Henan Cancer Hospital_English translation.pdf]

Medical Ethics Committee of Henan Cancer Hospital

Approval Letter of Ethics Review（Research Project）

Ethics Review No.：2019010

Project	Costs and quality of life associated with cervical cancer		
Principal investigator	Chen hongmin	Department	Department of Gynecological Oncology
Source of project	Researchers initiated		
Team Leader Unit	Chinese Academy of Medical Science	☐Single Centre, ☑Multicenter	
Team members	Hongmin Chen, Fangfang Rong, Manman Jia, Junli Deng		
Submissions	Protocol (Version number: NA; Date: 2018.10)<br>Informed consent(Version number: NA; Date: 2018.10)<br>Retrospective questionnaire<br>Prospective Questionnaire<br>Ethics approval of School of Public Health, Fudan University		

Key points for research design：

(Research purpose, objective and technology roadmap)

Purpose: Estimating costs associated with cervical cancer treatment and the heterogeneity across different developing regions in China from the perspective of family and total payer. Estimating health impact of cervical cancer among Chinese females.

Objectives：

1. Inclusion criteria in prospective design

Patients recruitment will be carried out in the study hospital. Cervical cancer outpatients and inpatients will be invited to participate in our study. Considering the general treatment process, we will recruit the following three categories of patients: (i) patients being admitted for cervical cancer treatment for the first time; (ii) patients being admitted for a new hospital spell, but not their first treatment; (iii) whether at the beginning of the treatment or halfway through the treatment, i.e. before or during two hospitalizations in the outpatient clinic.

2. Inclusion criteria in retrospective design

The following conditions were included in the study of cervical cancer patients admitted to the Henan Provincial Oncology Hospital in 2016: (1) aged 18 years or above; (2) without other cancers or precancerous; (3) with cervical cancers diagnosed based on pathological examination; (4) ever received or will receive inpatient treatment in the study hospitals.

3. Inclusion criteria for cross-sectional design of cervical cancer screening behavior.

Women aged 20-64, living in randomly-collected communities for one year or more, will be invited to participate in our survey.

Technology roadmap:

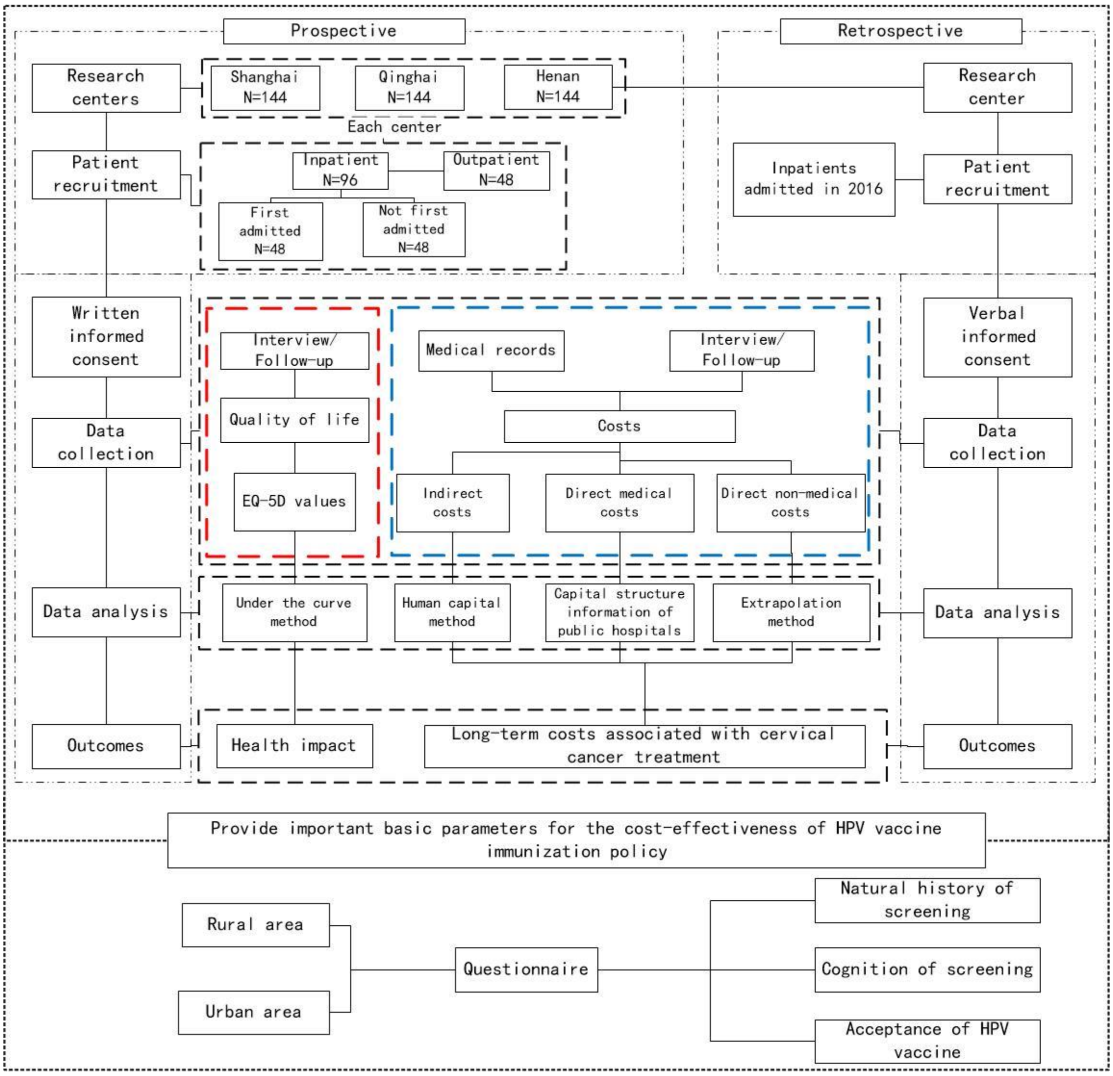

Does it involve biomedical information about human beings: ☑Yes， ☐No

(If not, skip the following 1-2 sections)

1. Type： ☑Medical records, ☐Specimen (☐Surplus ☐Collected accroding research design)

2. Research design :☑Retrospective, ☐Prospective (☑Observation ☐Intervention)

3. Informed consent measures:

This study is a non-interventional study. Participants will be fully informed of the research purpose, research procedure, risks, benefits and etc. Informed consent will be obtained from every paticipant.

4. Patients' privacy protection measures:

This study involves the confidentiality of patients' privacy information. Corresponding protection measures will be taken. When the research results are published publicly, the patient's disease privacy and information that may identify the patient will not be exposed.

Commitment and Review Application

The project team promises to carry out this research on the premise of following the requirements of relevant laws and regulations such as the declaration of Helsinki, the ethical review measures for biomedical research involving human beings, and following the principles of "informed consent, risk control, free and compensation, privacy protection, compensation

according to law, and special protection". The project team promises to register in the medical research registration system prior to the implementation of the study.			
Signature of applicant	Hongmin Chen	Date	2019.1.16
Ethical review			
According to the requirements of relevant laws and regulations such as the declaration of Helsinki issued by wma, the measures for ethical review of biomedical research involving human beings issued by the national health and Family Planning Commission, reviewed by the Ethics Committee:<br>☑Approval , ☐Amendment approved , ☐Revised review , ☐Disapproved			
Specific comments :			
☑ Rapid review, ☐Submitted to the Conference for review (Researchers need to report on site: ☐Yes, ☐No)			
Presiding committee member's statement	As a reviewer, there is no conflict of interest between me and the project.		
Signature of Presiding committee member	Jufeng Wang	Date	2019.1.21
Follow-up review frequency	12 months	Term of validity	
Tel: 0371-65588251; Address: No.127 Dongming Road, Zhengzhou			
Signature of Chairman (or Authorized Person)： Yongping Song Date： 2019.1.22			

Note: Positive and negative printing with a duplicate copy.
