# Supplementary material for: The economic burden of cervical cancer from diagnosis to one year after final discharge in Henan Province, China: A retrospective case series study
Source: PLoS One. 2020 May 7;15(5):e0232129. doi: 10.1371/journal.pone.0232129 (PMC7205285; doi:10.1371/journal.pone.0232129)
Supplement: S1 File — (ZIP) [file pone.0232129.s006.zip › Ethics Approvals/Ethics approval_Henan Cancer Hospital_origin.pdf]

河南省肿瘤医院医学伦理委员会

伦理审查批准函（科研项目）

伦理审查编号： 2018010

**项目名称**	宫颈癌的成本和生存质量研究		
**主要研究者**	陈红敏	**科室**	妇瘤科
**项目来源**	研究者发起		
**组长单位**	中国医学科学院		☐单中心，☑多中心
**项目组成员**	陈红敏、荣方方、贾漫漫、邓君丽		
**提交材料**	试验方案（版本号：NA；日期：2018 年 10 月）<br>知情同意书（版本号：NA；日期：2018 年 10 月）<br>回顾性调查问卷<br>前瞻性调查问卷<br>复旦大学公共卫生学院伦理批件		

研究设计要点：

（概述研究目的、对象、技术路线）

目的：从家庭和总支付人的角度估计中国不同发展水平的地区宫颈癌治疗的相关费用，以及宫颈癌对女性 HRQoL 的影响。

对象：

1.宫颈癌成本前瞻性设计的病例纳入

患者招募将在研究医院进行。宫颈癌门诊病人或住院病人将获邀参与我们的研究。考虑到该疾病的正常治疗过程，我们将会有以下三类患者:(i)首次住院接受宫颈癌治疗;(ii)接受新住院治疗，但不是首次治疗;(iii) 无论是在治疗开始时或治疗中途，即在住院前或两次住院期间在门诊接受诊疗的患者。

2.宫颈癌成本回顾性设计的病例纳入标准

从 2016 年河南省肿瘤医院收治的宫颈癌病人中，将符合以下条件的患者纳入研究：(1)年龄在 18 岁或以上；（2）没有其他癌症或癌前病变；（3）经病理检查诊断为宫颈癌患者；(4)曾在研究医院接受住院治疗。

3.宫颈癌筛查行为横断面设计的纳入标准

在随机抽取的社区内的妇女，年龄在 20-64 岁间，且在当地居住一年及以上的将被邀请参与我们的调查。

技术路线：

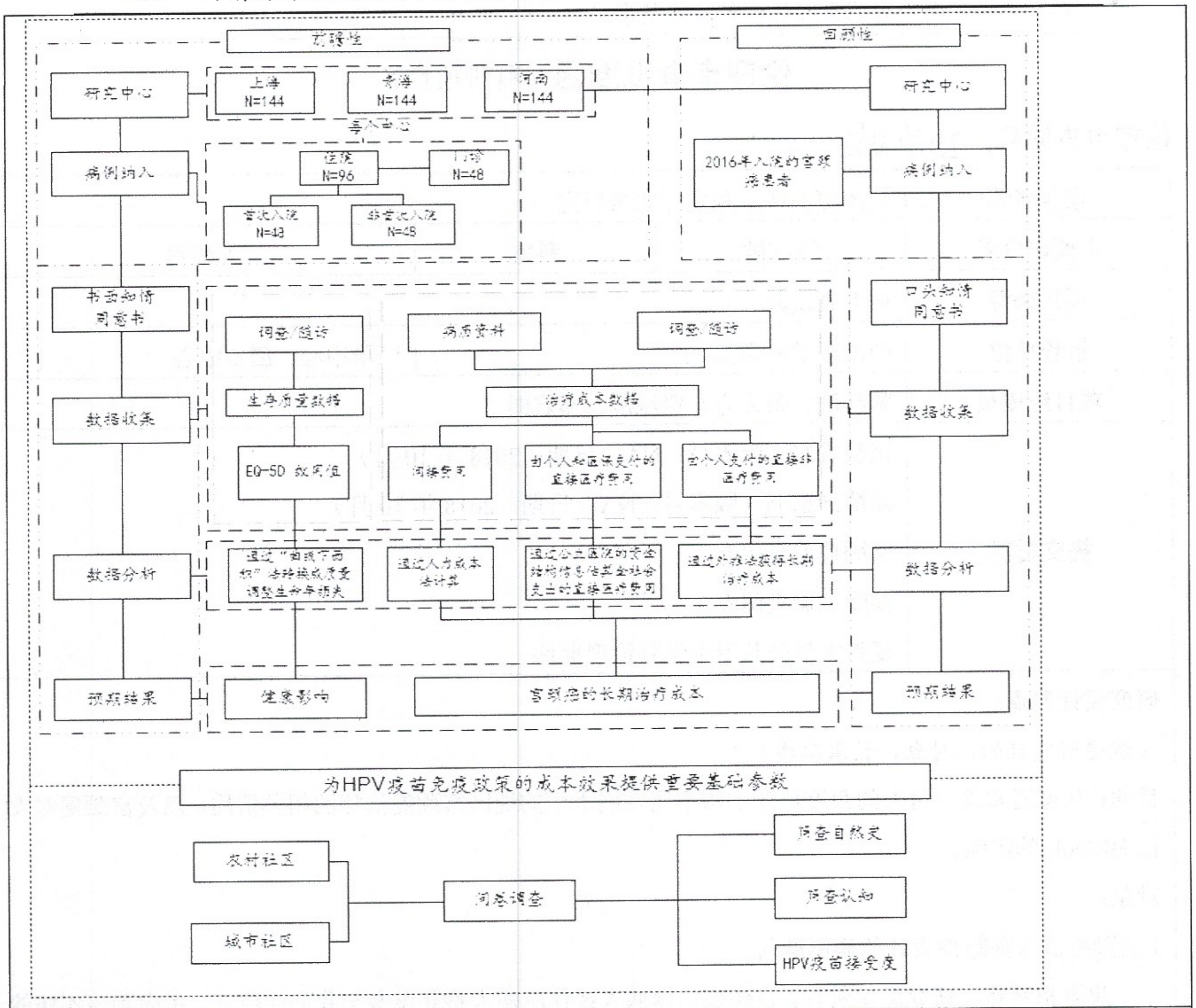

是否涉及人的生物医学信息：☑是，☐否（如否，跳过以下 1-2 部分）

1. 类型：☑病历；☐样本（☐剩余；☐按研究设计采集）

2. 研究设计：☑回顾性；☑前瞻性（☑观察性；☐实施干预）

3. 知情同意措施

本研究为非干预性研究，将充分告知患者研究目的、研究程序、风险与受益等，充分征得患者同意，签署知情同意书。

4. 患者隐私保护措施

涉及患者隐私信息保密，并有相应的保护措施。公开发表研究结果时，不会暴露患者疾病隐私及可能识别患者身份的信息。

承诺及审查申请

本项目组承诺在遵循《赫尔辛基宣言》、《涉及人的生物医学研究伦理审查办法》等相关法律法规要求的前提下开展此研究，遵循“知情同意原则，控制风险原则，免费和补偿原则，保护隐私原则，依

法赔偿原则，特殊保护原则”。承诺在实施研究前在医学研究登记备案信息系统进行登记。			
申请人签名	[illegible]	日期	2019.1.16
伦理审查意见			
依据 WMA《赫尔辛基宣言》、国家卫生计生委《涉及人的生物医学研究伦理审查办法》等相关法律法规的要求，经本伦理委员会审查：<br>☑批准，□修改后批准，□修改后再审，□不批准			
具体意见：			
☑快速审查，□提交会议审查（是否需要研究者到场汇报：□是，□否）			
主审委员声明	作为审查人员，我与该项目之间不存在相关利益冲突		
主审签名	[illegible]	日期	2019.1.21
跟踪审查频率	12 个月	有效期	
联系方式：0371-65588251，郑州市东明路 127 号			
主任委员（或授权者）签字：[illegible]		日期：2019.1.22	

河南省肿瘤医院医学伦理委员会

注：正反打印，一式两份。
