# Supplementary material for: The economic burden of cervical cancer from diagnosis to one year after final discharge in Henan Province, China: A retrospective case series study
Source: PLoS One. 2020 May 7;15(5):e0232129. doi: 10.1371/journal.pone.0232129 (PMC7205285; doi:10.1371/journal.pone.0232129)
Supplement: S1 File — (ZIP) [file pone.0232129.s006.zip › Ethics Approvals/Ethics approval_School of Public Health, Fudan University.pdf]

復旦大學 公共卫生学院
School of Public Health Fudan University
上海市东安路130号 邮编 200032 130 Dong An Road, Shanghai 200032, P.R.China http//sph.fudan.edu.cn Email: fdsph@fudan.edu.cn

研究课题： 宫颈癌的成本和生存质量研究

课题负责人： 钱梦岑 博士 复旦大学公共卫生学院

复旦大学公共卫生学院医学研究伦理委员会于 2018 年 10 月 30 日批准了钱梦岑博士有关《宫颈癌的成本和生存质量研究》的伦理学申请。批准号为 IRB#2018-10-0710，有效期为即日起至 2020 年 1 月 31 日。

在项目开展期间，研究方案及具体工作计划、调查表、知情同意书等如有任何修改和变动，必须在向本委员会报告并获得批准后方可付诸实施；由于研究项目的执行对研究对象造成的不良影响或后果必须向本委员会报告并征求调查指导。研究项目在有效期后还将继续进行的，必须向本委员会申请批准延期。

复旦大学公共卫生学院医学研究伦理委员会

国际注册号：IRB00002408 & FWA00002399

2018 年 10 月 30 日

復旦大學 公共卫生学院
School of Public Health Fudan University
上海市东安路130号 邮编 200032 130 Dong An Road, Shanghai 200032, P.R.China http//sph.fudan.edu.cn Email: fdsph@fudan.edu.cn

Notice of Action

Fudan University School of Public Health
Institutional Review Board (IRB)

Principal Investigator: Dr. Mengcen Qian

Protocol Title: Costs and Quality of Life associated with Cervical Cancer

IRB Approval Number: **IRB #2018-10-0710** (Contact: 86-21-54237051)

Committee Action: **Approved on**: Oct 30 , 2018 **Expires**: Jan 31 , 2020

We have received the information you sent regarding the above named protocol. This information complies with the modifications required by the Institutional Review Board, and your protocol is now approved. You may begin collecting data at any time.

Our IRB is registered with the Office for Human Research Protections, IRB00002408, and has a Federal wide Assurance, FWA00002399.

Thank you for your cooperation.

Qingwu, Jiang
Chair of Fudan University IRB#1
P. R. China

復旦大學 公共卫生学院
School of Public Health Fudan University
上海市东安路130号 邮编 200032 130 Dong An Road, Shanghai 200032, P.R.China http//sph.fudan.edu.cn Email: fdsph@fudan.edu.cn

INVESTIGATOR RESPONSIBILITIES:

All copies of the informed consent form must be made from this approved version. Any changes to the consent form must be approved in advance by the IRB.

Any changes to the protocol must be approved by the IRB before they are implemented.

Any new information that would affect potential risks to subjects or any adverse reactions must be reported immediately to the IRB contact listed above.

If the research will continue beyond the expiration date indicated above, a request for renewal/continuing review must be submitted to the IRB. You must obtain approval **before** the current expiration date. If you do not obtain approval by the expiration date noted above, you are not authorized to collect any data until the IRB re-approves your protocol.

Signed consent forms must be retained in study site for three years following the end of the project.
